# Functional genomic investigation of *CYP71AJ49* in *Peucedanum praeruptorum* Dunn: *Agrobacterium*-mediated overexpression reveals dual roles in drought adaptation and coumarin biosynthesis

**DOI:** 10.3389/fpls.2025.1707087

**Published:** 2025-12-17

**Authors:** Maosuo Wang, Shenghua Wang, Huifang Chu, Qian Ruan, Jun Dai, Peipei Wei

**Affiliations:** 1 College of Biological and Pharmaceutical Engineering, West Anhui University, Lu’an, Anhui, China; 2 Engineering Technology Research Center of Plant Cell, West Anhui University, Lu’an, Anhui, China

**Keywords:** *Peucedanum praeruptorum* Dunn, CYP71AJ49, drought stress, transgenic *Arabidopsis*, coumarins, transcriptome

## Abstract

**Introduction:**

Drought stress is one of the major limiting factors for plant growth and agricultural production worldwide. This study seeks to investigate the role and mechanism of CYP71AJ49, a key enzyme gene in the coumarin biosynthesis pathway, under drought stress, and to elucidate its dual functions in drought tolerance and coumarin synthesis.

**Methods:**

CYP71AJ49 was isolated and cloned from Peucedanum praeruptorum Dunn, and heterologously overexpressed in Arabidopsis thaliana. Drought stress was mimicked with 20% PEG, after which transcriptome sequencing, analysis of physiological and biochemical parameters (including root length, fresh weight, antioxidant enzyme activities, proline, and malondialdehyde), and qRT-PCR validation were performed. Furthermore, coumarin content was quantified using ultra-performance liquid chromatography.

**Results:**

Our findings demonstrated that overexpression of CYP7IAJ49 remarkably improved the drought tolerance of Arabidopsis thaliana. Under drought stress, transgenic plants exhibited significant increases in root length (22.84%), fresh weight (164.91%), and water content (294.41%). Simultaneously, their carbohydrate metabolism pathway was activated, the transcription levels of stress-responsive genes were upregulated, antioxidant enzyme activities and proline content were significantly enhanced, while malondialdehyde content was substantially decreased. Furthermore, CYP7IAJ49 also significantly promoted the accumulation of bergapten (52.42%) in Arabidopsis thaliana.

**Discussion:**

Our findings demonstrate that CYP71AJ49 improves plant drought resistance through multiple pathways while regulating the accumulation of coumarin secondary metabolites. This discovery not only expands our understanding of the functions of CYP450 family genes but also provides critical theoretical support and genetic resources for breeding new crop varieties with high drought tolerance and high coumarin content.

## 1 Introduction

The CYP71 gene family, part of the cytochrome P450 (CYP450) superfamily, represents one of the largest subfamilies in plants. It performs a pivotal function in the synthesis and accumulation of plant secondary metabolites, and it has drawn considerable research attention in recent years (Nie et al., 2024). With ongoing investigations by researchers worldwide, studies have revealed that the *CYP71AJ49* gene in *Peucedanum praeruptorum* Dunn (*P. praeruptorum*) fulfills a pivotal function within the coumarin biosynthesis pathway. Specifically, this gene encodes a protein acting as a psoralen synthase, which specifically catalyzes the conversion of marmesin to psoralen (Jian et al., 2020). The roots of *P. praeruptorum* are used medicinally, and the traditional Chinese medicine “Qianhu” refers to the dried roots of this plant (Chinese Pharmacopoeia Commission, 2020). In China, it has been used as a medicinal herb for over a thousand years (Xie et al., 2023). Modern chemical analyses have identified coumarin compounds as the main active constituents of *P. praeruptorum*, including simple coumarins, furanocoumarins, and pyranocoumarins (Du et al., 2022). Coumarins, which are phenolic secondary metabolites with properties such as plant growth regulation, antioxidant activity, and antimicrobial effects, have been proven to perform a vital function in plant defense against external stresses (Saleh and Madany, 2015; Cui et al., 2019; Ahanger et al., 2020). For example, scopoletin is associated with scavenging reactive oxygen species and pathogen defense (Döll et al., 2018), while psoralen exhibits anti-inflammatory properties and contributes significantly to plant infection resistance (Walker et al., 2012; Zhang et al., 2019b).

According to the central dogma, the accumulation of metabolites is regulated by genes involved in their biosynthetic pathways. Thus, the expression levels of key enzyme genes in the coumarin biosynthetic pathway often profoundly influence both the accumulation of coumarins and stress resistance in plants. For instance, Duan et al. (2019) revealed that the *GmF6’H1* gene, which encodes an enzyme in the coumarin synthesis pathway, enhances stress tolerance in transgenic *Arabidopsis thaliana* (*Arabidopsis*) by elevating endogenous coumarin accumulation. In a similar vein, Jian et al. (2020) observed that the transcript levels of *CYP71AJ49*, a pivotal gene encoding a critical enzyme in coumarin biosynthesis in *P. praeruptorum*, undergo significant alterations under abiotic stresses such as ultraviolet radiation, low, and high temperature. Researchers have hypothesized that the expression of *CYP71AJ49* could modulate the expression of stress-responsive genes and act in synergy with other core enzymes within the coumarin biosynthetic pathway, including *PpPAL*, *Pp4CL*, and *PpC2’H*, thereby promoting coumarin accumulation in plant tissues and enhancing tolerance to abiotic stress.

Drought stress is considered to be among the most significant abiotic factors that impede plant growth and development. Previous studies have indicated that key physiological systems in plants, including photosynthesis, antioxidant capacity, and osmotic regulation, are substantially compromised under drought conditions (Ihsan et al., 2016; Dong et al., 2022). For instance, under polyethylene glycol 6000 (PEG) induced drought stress, sorghum seeds exhibit marked suppression of radicle, hypocotyl, and plumule growth due to the strongly negative osmotic potential (Abreha et al., 2021). To mitigate drought-induced oxidative damage, plants activate adaptive mechanisms by regulating stress-responsive genes to enhance their antioxidant capacity and osmotic adjustment (Hura et al., 2022). In *Arabidopsis*, for instance, the upregulation of antioxidant enzyme genes such as *AtCAT*, *AtPOD*, and *AtSOD*, along with increased enzymatic activities, strengthens the antioxidant system and alleviates excessive reactive oxygen species (ROS) accumulation caused by drought stress (Esmaeili et al., 2019; Mehari et al., 2022; Yu et al., 2023). In the investigation conducted by Wang et al. (2021), parameters including water loss rate, ROS levels, and malondialdehyde (MDA) content were used as key indicators to evaluate the enhanced drought tolerance conferred by *EjWRKY17* in *Arabidopsis*.

Beyond the antioxidant and osmoregulatory systems, plant responses to drought involve the coordinated regulation of multiple genes and metabolic pathways (Seki et al., 2001). For example, Wang et al. (2024) conducted transcriptomic analyses of drought-tolerant and drought-sensitive *Chenopodium quinoa Willd* genotypes and found that differentially expressed genes (DEGs) were significantly enriched in starch and sucrose metabolism pathways. Furthermore, various secondary metabolites derived from the phenylpropanoid pathway, particularly coumarin compounds, demonstrate notable antioxidant activity under abiotic stress conditions (Xia et al., 2023; Saini et al., 2024). In recent years, the relationship between coumarins and abiotic stress has gained increasing research attention. For instance, Nie et al. (2024) demonstrated that the *MaCYP82L1* gene in *Melilotus albus* not only conferred enhanced drought resistance in heterologously expressed yeast but also increased coumarin content in transgenic hairy roots. However, whether overexpression of the key coumarin biosynthetic gene *CYP71AJ49* from *P. praeruptorum* in *Arabidopsis* improves drought tolerance and coumarin accumulation remains to be thoroughly investigated.

In the present study, the key enzyme gene *CYP71AJ49* from the coumarin biosynthetic pathway was cloned from *P. praeruptorum* and heterologously expressed in *Arabidopsis* to examine its functional role in drought stress response and its effect on regulating coumarin biosynthesis. To investigate the gene function under drought stress, we evaluated a range of physiological parameters in the overexpression lines, including leaf water loss rate, chlorophyll content, antioxidant enzyme activities, and coumarin content. These physiological assessments were integrated with transcriptome sequencing and quantitative real-time polymerase chain reaction (qRT-PCR) analyses. This study provides the first evidence of the dual functional role of *CYP71AJ49* in mediating drought stress response and regulating coumarin metabolism. These findings not only offer novel insights into the mechanistic link between abiotic stress adaptation and coumarin biosynthesis in plants but also facilitate the development of new crop varieties with enhanced drought tolerance and elevated coumarin content through molecular breeding strategies.

## 2 Materials and methods

### 2.1 Plant materials and treatments

The seeds of *P. praeruptorum* and wild-type (WT) *Arabidopsis* (Columbia ecotype) stored in our laboratory were employed for this research. For seeds, surface sterilization was performed using 75% ethanol for 30 seconds, followed by thorough rinsing nine times with sterile water, and finally sown onto 1/2 MS solid medium. Before sowing, seeds were stratified at 4°C for 36 hours for vernalization, then transferred to a growth incubator at 20°C for germination. After germination, uniformly grown seedlings were selected and transplanted into pots filled with sterilized vermiculite for subsequent cultivation in a phytotron.

### 2.2 Cloning of the *CYP71AJ49* gene

Total RNA was isolated from *P. praeruptorum* seedlings propagated in our laboratory using the protocol described by Wei et al. (2023), followed by reverse transcription into complementary DNA (cDNA). This cDNA functioned as a template to amplify the full-length *CYP71AJ49* gene sequence using PCR. The resulting PCR products were purified and submitted for sequencing. The obtained sequence was aligned with the *CYP71AJ49* gene (GenBank: MN010766.1) sequence in the National Center for Biotechnology Information (NCBI) database (**Supplementary Figure S1**). After confirmation, the gene fragment was constructed into the plant expression vector pCAMBIA1300 (p1300) via homologous recombination. Among them, homologous recombination primers include additional *Xba* I and *Bam*H I restriction sites. The recombinant plasmid underwent verification through restriction enzyme digestion and PCR. The correctly assembled construct was then transformed into competent cells of *Agrobacterium tumefaciens* GV3101 using the heat shock method, to support subsequent transgenic procedures.

### 2.3 Overexpression of the *CYP71AJ49* gene in *Arabidopsis*

The recombinant vector p1300-CYP71AJ49 was integrated into the *Arabidopsis* genome via the floral dip method (Clough and Bent, 1998), followed by the collection of T1 seeds. Positive transformants were screened on medium supplemented with 30 μg/mL hygromycin B. Genomic DNA was extracted from transgenic lines and further confirmed by PCR. After continuous cultivation and selection, homozygous T3 transgenic *Arabidopsis* lines (CYP71AJ49-OE) were obtained. Three representative transgenic lines (OE-1, 2, 3) were selected for subsequent experiments.

### 2.4 RNA extraction, library construction, sequencing, and assembly

WT and CYP71AJ49-OE plant samples treated with 20% PEG for 4 hours were collected. TRIzol reagent was used to extract total RNA. The purity of the RNA was determined via a spectrophotometer, and integrity was evaluated using an Agilent 5400 Fragment Analyzer system. Oligo(dT) magnetic beads were used to enrich poly(A)+ mRNA following the protocol provided with the NEB Next Ultra II RNA Library Prep Kit for Illumina (New England Biolabs Inc., MA, USA). The mRNA was fragmented to approximately 300 bp for cDNA synthesis. Subsequent to end repair, 3′-terminal adenylation, and adapter ligation, cDNA fragments of about 300 bp were isolated with AMPure XP beads, subjected to PCR amplification, and purified to construct the sequencing library.

Library quantification was conducted with a Qubit 2.0 Fluorometer, insert size was analyzed via the Agilent 5400 system, and effective concentration was accurately determined via qRT-PCR. Finally, paired-end sequencing was carried out on`12 the Illumina Novaseq™ 6000 platform.

### 2.5 Identification of DEGs and enrichment analysis

DESeq2 was used to analyze transcriptome data from WT and CYP71AJ49-OE plants. DEGs were identified using thresholds of *p* < 0.05 and |log2fc|≥ 1. Volcano plots were used to visualize DEGs expression levels. To examine biological functions, Gene Ontology (GO) functional annotation and Kyoto Encyclopedia of Genes and Genomes (KEGG) pathway enrichment analyses were carried out. Statistical analysis and visualization were conducted using SPSS v26.0, clusterProfiler v4.6, ggplot2 v3.5, and GraphPad Prism v10.4.

### 2.6 qRT-PCR analysis of stress-responsive gene expression in *Arabidopsis*

WT and CYP71AJ49-OE plants were harvested at 0, 2, 4, 8, 12, and 24 hours after PEG treatment. Subsequent to this, total RNA was extracted, reverse-transcribed into cDNA, and the resulting cDNA was stored at –80°C. Using cDNA as template, qRT-PCR was performed with 2× SYBR Green qPCR Mix (Coolaber, Beijing, China) on a QuantStudio 5 Real-Time PCR System (San Jose, CA, USA). The expression levels of stress-related genes (*AtKT1*, *AtNHX1*, *AtAVP1*, *AtMnSOD*, *AtPOD*, *AtAPX1*, and *AtP5CS2*) were analyzed. Primers were designed using NCBI’s database resources, and all primer sequences employed in this study are detailed in the **Supplementary Table S1**. *AtACT2* served as the internal reference gene, and the 2^−ΔΔCT^ method was used to determine relative gene expression (Schmittgen and Livak, 2008). The GenBank or RefSeq accessions of stress-responsive *Arabidopsis* genes are provided in **Supplementary Table S2**.

### 2.7 Physiological and biochemical measurements

We collected fresh leaves from WT and CYP71AJ49-OE plants, with their weight recorded as the initial weight (0 h). Then, under room temperature conditions, we reweighed these same leaves at 0.5, 1, 2, 3, 4, and 5 hours post-initial weighing, and these weights were used to calculate the water loss rate. The formula for calculating leaf water loss rate is as follows: (initial mass - mass at the target time point)/initial mass × 100%. *Arabidopsis* plants treated with 20% PEG for 10 days were designated as the experimental group, while non-treated plants served as the control group. Subsequent to the measurement of the fresh weight and maximum root length of the experimental and control groups, the plants were expeditiously transferred to an oven at 105°C for 15 minutes for inactivation. Thereafter, they were desiccated to constant weight at 60°C, and the dry weight was measured. Water content was calculated as (fresh weight – dry weight)/fresh weight × 100%. Chlorophyll content was determined using the acetone extraction method (Arnon, 1949). Additionally, in accordance with the manufacturer’s instructions, the levels of superoxide dismutase (SOD), peroxidase (POD), catalase (CAT), ascorbate peroxidase (APX), total glutathione (T-GSH), MDA, and proline (Pro) were measured in both the experimental and control groups using a commercial kit (Coolaber, Beijing, China).

### 2.8 Coumarin content measurement

CYP71AJ49-OE and WT plant samples underwent drying in a 37°C constant-temperature oven for 48 hours, ground into powder, and screened through an 80-mesh sieve. Accurately weigh 0.3 g of the powder and dilute to a final volume of 25 mL with methanol. The extraction was carried out by subjecting the sample to ultrasonication at 250 W and 33 kHz for 30 minutes. After cooling, the volume was readjusted. After being filtered through a 0.22 μm organic filter membrane, the supernatant was analyzed by Ultra-Performance Liquid Chromatography (UPLC). Chromatographic separation was performed on an Agilent ZORBAX RRHD Eclipse Plus 95A C18 column (100 mm × 2.1 mm; 1.8 μm) with water (A) and methanol (B) as mobile phases under the gradient elution program listed in **Supplementary Table S3**. The injection volume was set at 2 μL, the column temperature was 35°C, the flow rate was maintained at 0.25 mL/min, and the detection wavelength was adjusted to 321 nm.

### 2.9 Statistical analysis

With SPSS v26.0 software, statistical analysis was carried out. Experimental results are shown as mean ± standard deviation from 3 biological replicates. Comparisons between groups were conducted using one-way analysis of variance or Tukey’s HSD test, with a significance threshold established at *p* < 0.05.

## 3 Results

### 3.1 Overexpression of the *CYP71AJ49* gene in *Arabidopsis*

**Figure 1:**
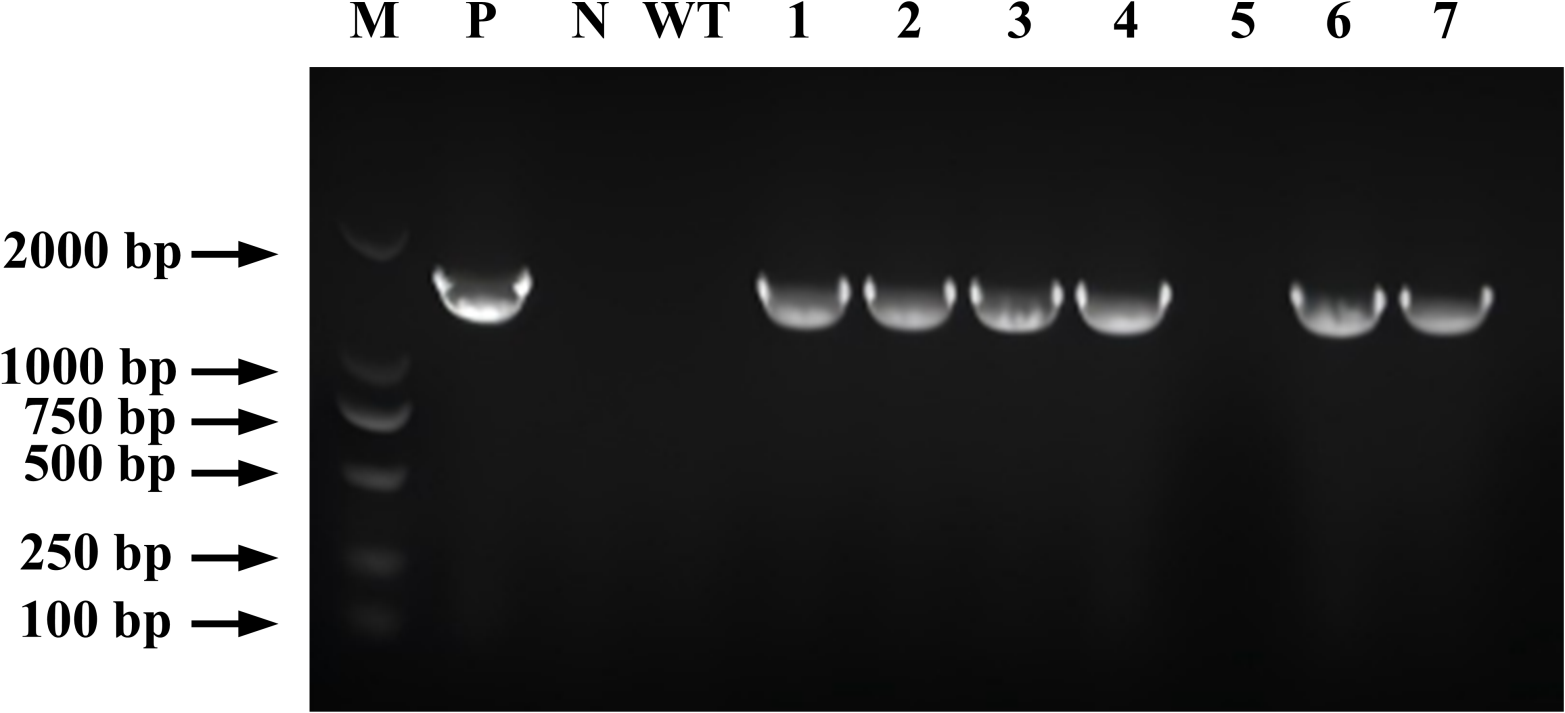
PCR identification of *CYP71AJ49* transgenic *Arabidopsis*. M is DL2,000 DNA Marker; P is the positive control; N is the negative control; WT is the wild-type *Arabidopsis*; 1–7 are transgenic *Arabidopsis*.

To verify the integration of the *CYP71AJ49* gene in various transgenic lines, PCR validation was performed using DNA extracted from T1 generation CYP71AJ49-OE *Arabidopsis* leaves. Agarose gel electrophoresis showed that the target band matched the size of the positive control band (**Figure 1**), indicating successful integration of the *CYP71AJ49* gene into the *Arabidopsis* genome. A total of six positive transgenic seedlings were obtained, along with one false-positive plant. The complete scan image of the agarose gel is presented in **Supplementary Figure S2**.

### 3.2 Effect of *CYP71AJ49* gene overexpression on drought resistance of *Arabidopsis*

**Figure 2:**
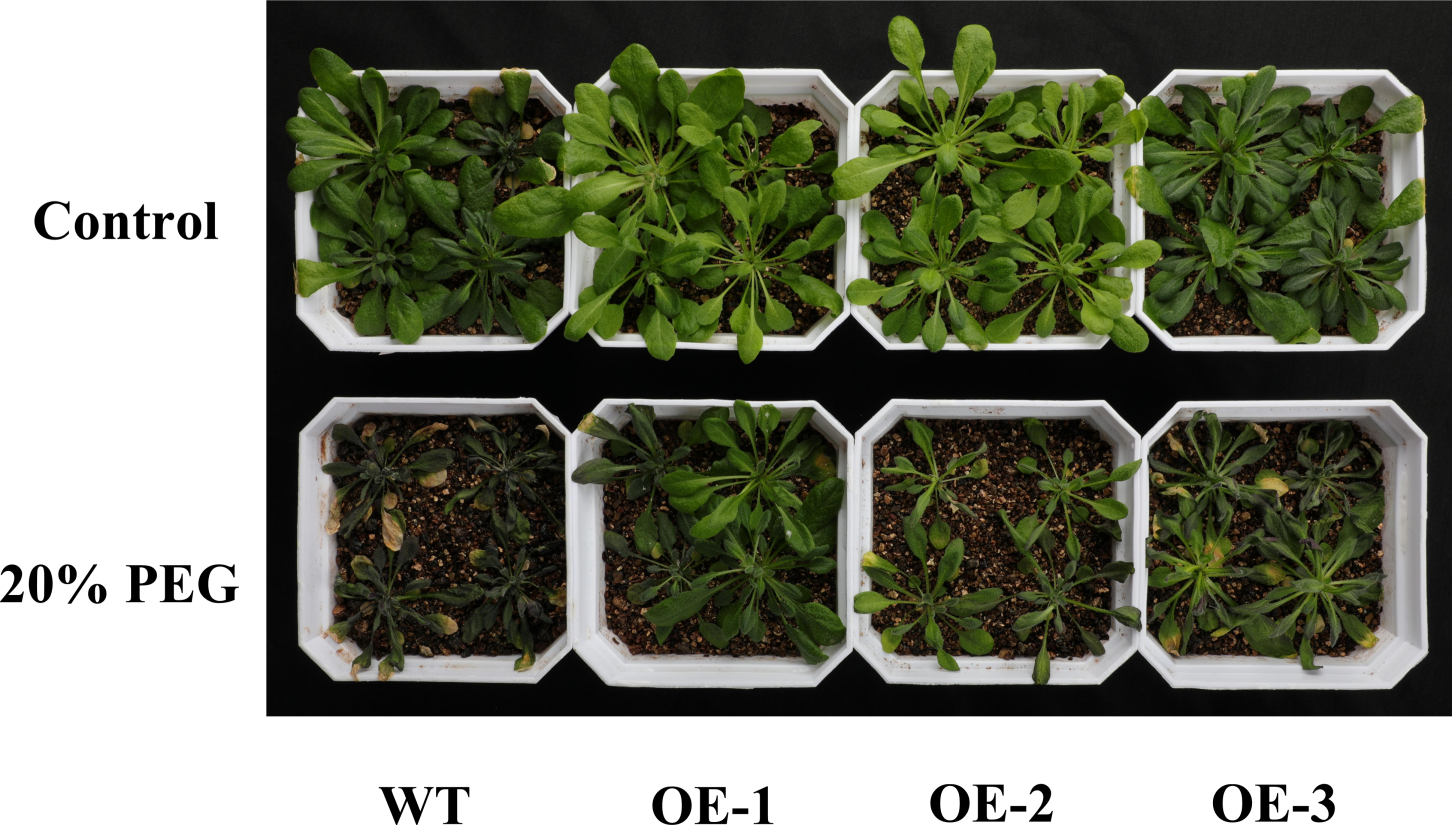
Phenotypic comparison of WT and *CYP71AJ49*-overexpressing transgenic *Arabidopsis* under drought stress. Significant phenotypic differences between WT and transgenic *Arabidopsis* lines were observed after 10 days of treatment with 20% PEG.

**Figure 3:**
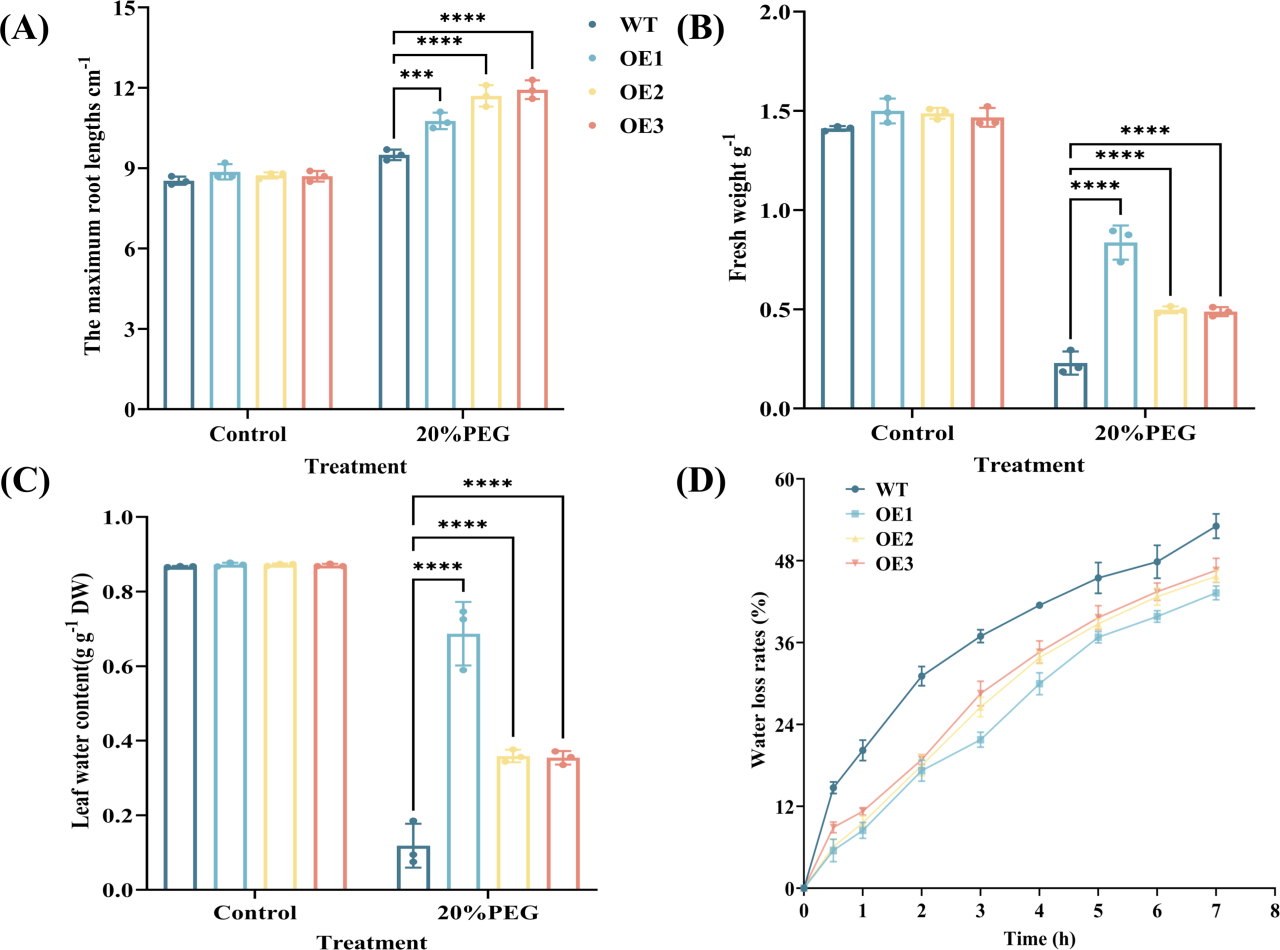
Effects of 20% PEG-induced drought stress on physiological parameters in *CYP71AJ49*-overexpressing *Arabidopsis*. **(A)** The maximum root lengths; **(B)** Fresh weight; **(C)** Leaf water content; **(D)** Water loss rates. The vertical error bars in the figures denote means ± SD (n = 3) and asterisks indicate statistically significant (***p < 0.001; ****p < 0.0001 based on Tukey’s HSD test).

Under normal growth conditions, no obvious phenotypic differences were noted between WT and CYP71AJ49-OE plants. After 10 days of treatment with 20% PEG, WT plants exhibited more severe leaf yellowing and wilting compared to CYP71AJ49-OE plants (**Figure 2**). Under drought stress, maximum root length, fresh weight, and leaf water content were significantly higher in CYP71AJ49-OE plants than in WT, with average increases of 22.84%, 164.91%, and 294.41% (**Figures 3A–C**), respectively. Additionally, the water loss rate was significantly lower in CYP71AJ49-OE plants, showing an average reduction of 30.65% at 3 hours post-treatment (**Figure 3D**).

### 3.3 Transcriptome sequencing and assembly

To further explore the molecular mechanisms underlying drought tolerance enhancement in *Arabidopsis* by *CYP71AJ49* overexpression, RNA-seq analysis was performed on WT and CYP71AJ49-OE plants treated with 20% PEG for 4 hours. Twelve cDNA libraries were constructed in total, yielding 90.54 gigabytes (Gb) of clean data. The Q20 values for all samples exceeded 99.49%. The average clean data per library reached 50.37 million reads, with clean bases ranging from 6.15 Gb to 8.80 Gb per sample. The percentages of Q20 and Q30 bases ranged from 99.49% to 99.59% and from 97.95% to 98.36%, respectively, and the GC content varied from 44.82% to 45.54%, indicating high sequencing quality. The alignment rate of clean reads to the reference genome exceeded 98.16% for all samples, confirming the high reliability of data assembly (**Supplementary Table S4**).

### 3.4 Differentially expressed genes analysis

**Figure 4:**
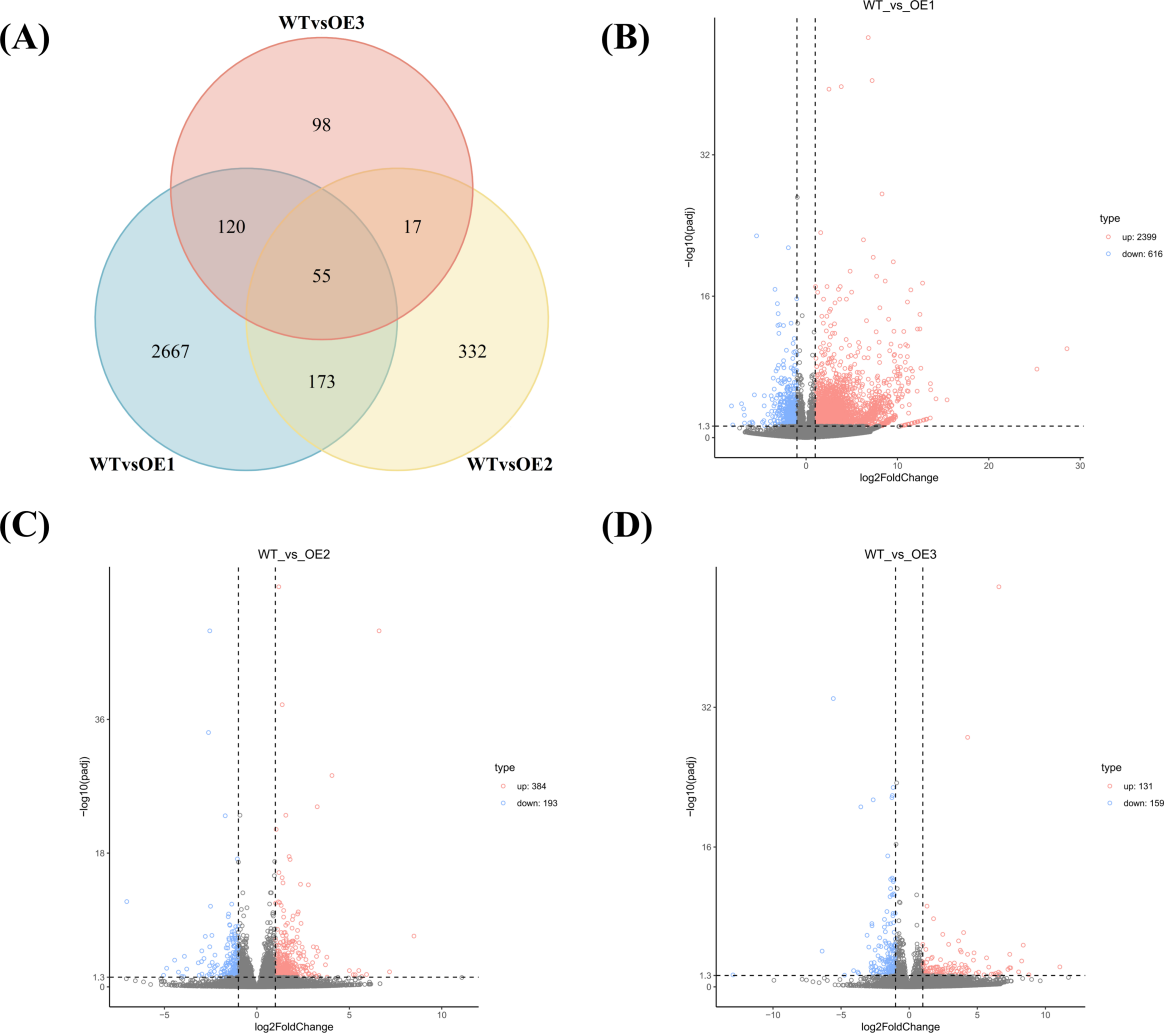
DEGs analysis of drought stress response in *CYP71AJ49*-Overexpressing and WT *Arabidopsis*. **(A)** Venn diagram. Volcano plot of DEGs: **(B)** WTvsOE1; **(C)** WTvsOE2; **(D)** WTvsOE3.

A total of 3,882 DEGs were detected in the three CYP71AJ49-OE lines (OE-1, OE-2, and OE-3) when compared to WT. Among these, 55 DEGs were common to three lines (**Figure 4A**). Specifically, between WT and OE-1, 3,015 DEGs were identified, comprising 636 up-regulated and 1,950 down-regulated genes; between WT and OE-2, 577 DEGs, with 384 up-regulated and 193 down-regulated; and between WT and OE-3, 290 DEGs, including 131 up-regulated and 159 down-regulated genes (**Figures 4B–D**).

### 3.5 GO and KEGG enrichment analyses

**Figure 5:**
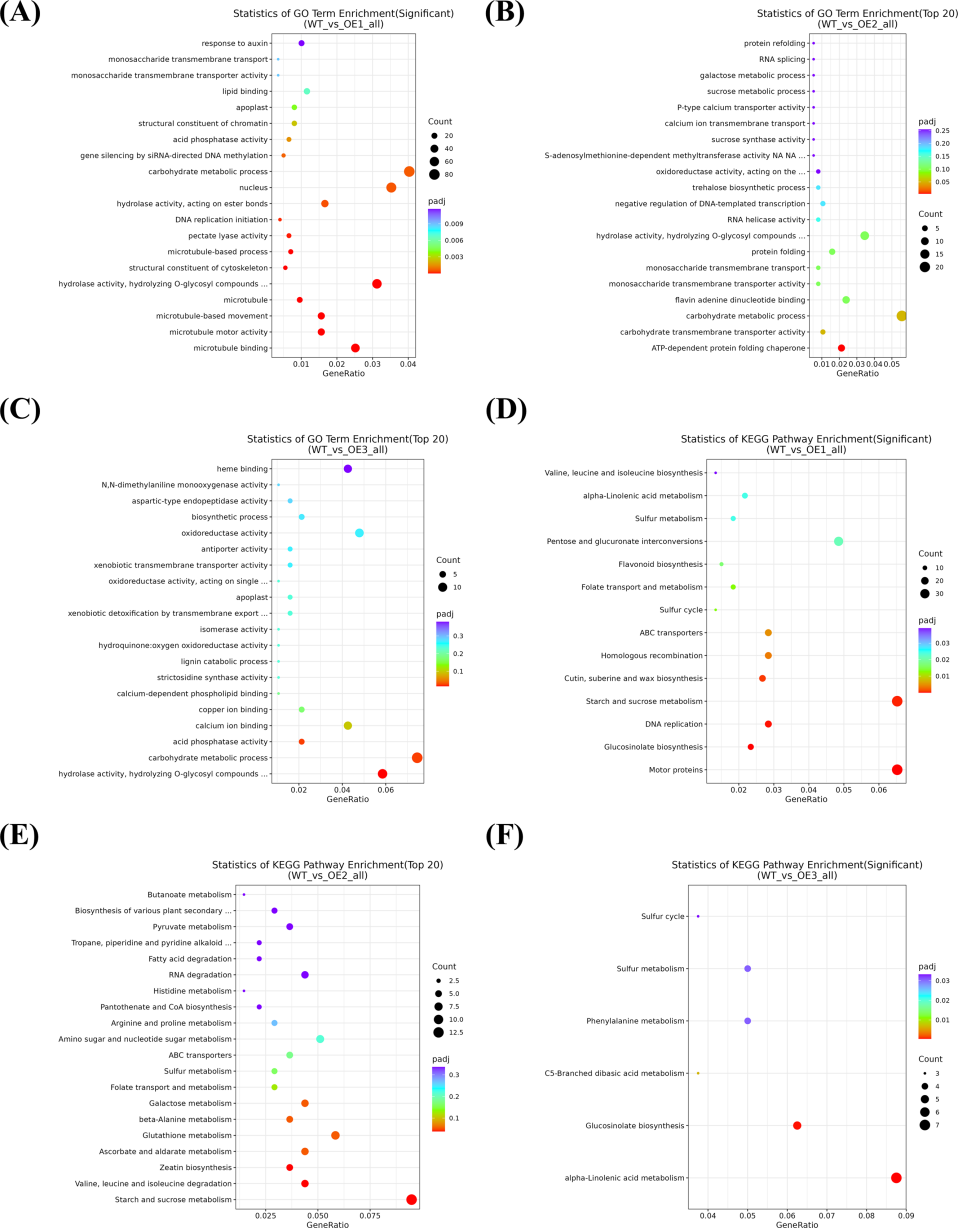
GO functional annotation and KEGG pathway enrichment analysis of DEGs between WT and *CTP71AJ49*-overexpressing *Arabidopsis* under drought stress. **(A)** GO analysis of DEGs in WTvsOE1; **(B)** GO analysis of DEGs in WTvsOE2; **(C)** GO analysis of DEGs in WTvsOE3; **(D)** KEGG pathway analysis of DEGs in WTvsOE1; **(E)** KEGG pathway analysis of DEGs in WTvsOE2; **(F)** KEGG pathway analysis of DEGs in WTvsOE3.

For a deeper exploration of the molecular mechanisms underlying improved drought tolerance mediated by *CYP71AJ49*, GO functional and KEGG pathway enrichment analyses were conducted on the DEGs. Under drought stress, comparisons between WT and each transgenic line were significantly enriched in GO terms, including hydrolase activity, hydrolyzing O-glycosyl compounds, carbohydrate metabolic process, monosaccharide transmembrane transport, acid phosphatase activity, monosaccharide transmembrane transporter activity, and apoplast, with carbohydrate metabolic process and hydrolase activity, hydrolyzing O-glycosyl compounds being the most prominent (**Figures 5A–C**). KEGG analysis revealed that 598, 137, and 80 DEGs were annotated in WTvsOE-1, WTvsOE-2, and WTvsOE-3, respectively, involved in 124, 91, and 64 pathways (**Figures 5D–F**). The starch and sucrose metabolism pathway showed the highest enrichment of DEGs, indicating its crucial role in the *CYP71AJ49*-mediated drought stress response.

### 3.6 Effect of *CYP71AJ49* overexpression on stress-responsive gene expression in *Arabidopsis*

**Figure 6:**
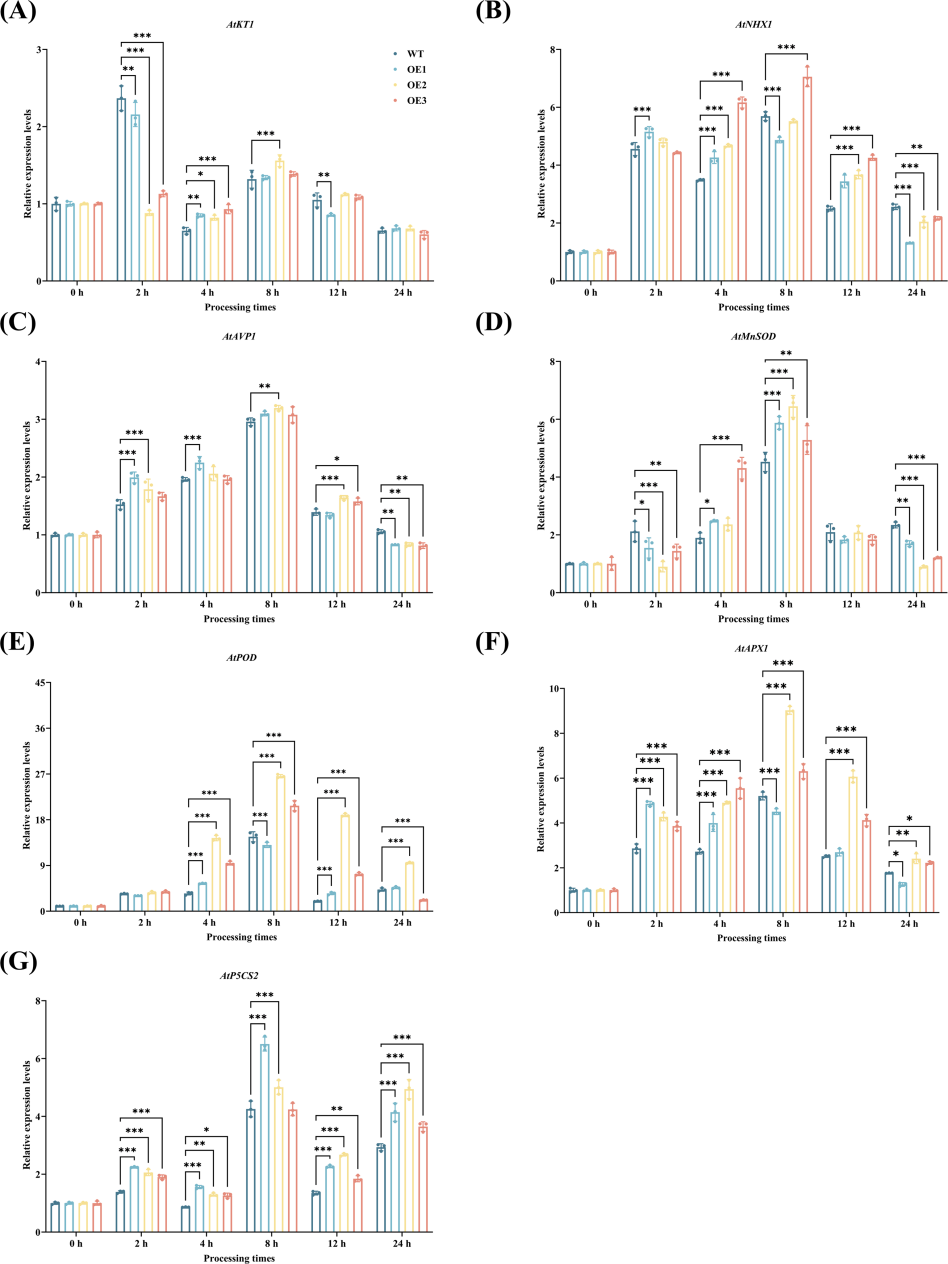
Effects of 20% PEG-induced drought stress on the relative expression levels of stress-responsive genes in *CYP71AJ49*-overexpressing transgenic *Arabidopsis*. **(A)***AtKT1*; **(B)***AtNHX1*; **(C)***AtAVP1*; **(D)***AtMnSOD*; **(E)***AtPOD*; **(F)***AtAPX1*; **(G)***AtP5CS2*. The vertical error bars in the figures denote means ± SD (n = 3) and asterisks indicate statistically significant (*p < 0.05; **p < 0.01; ***p < 0.001 based on Tukey’s HSD test).

To further investigate the molecular mechanism by which the *CYP71AJ49* gene enhances plant drought resistance, we screened out genes closely related to drought stress response based on transcriptome analysis and literature review. After 20% PEG treatment, the expression levels of stress-responsive genes (*AtKT1*, *AtNHX1*, *AtAVP1*, *AtMnSOD*, *AtPOD*, *AtAPX1*, and *AtP5CS*2) in the WT and CYP71AJ49-OE plants alike generally increased initially and then decreased (**Figure 6**). Compared to WT, the expression of most stress-responsive genes was up-regulated in CYP71AJ49-OE lines, with OE-2 showing the most significant enhancement. Most genes reached peak expression at 8 hours post-stress. Notably, the osmoregulatory gene *AtP5CS2* exhibited a different expression pattern, with a second rise at 24 hours.

### 3.7 Effect of *CYP71AJ49* overexpression on antioxidant capacity under drought stress

**Figure 7:**
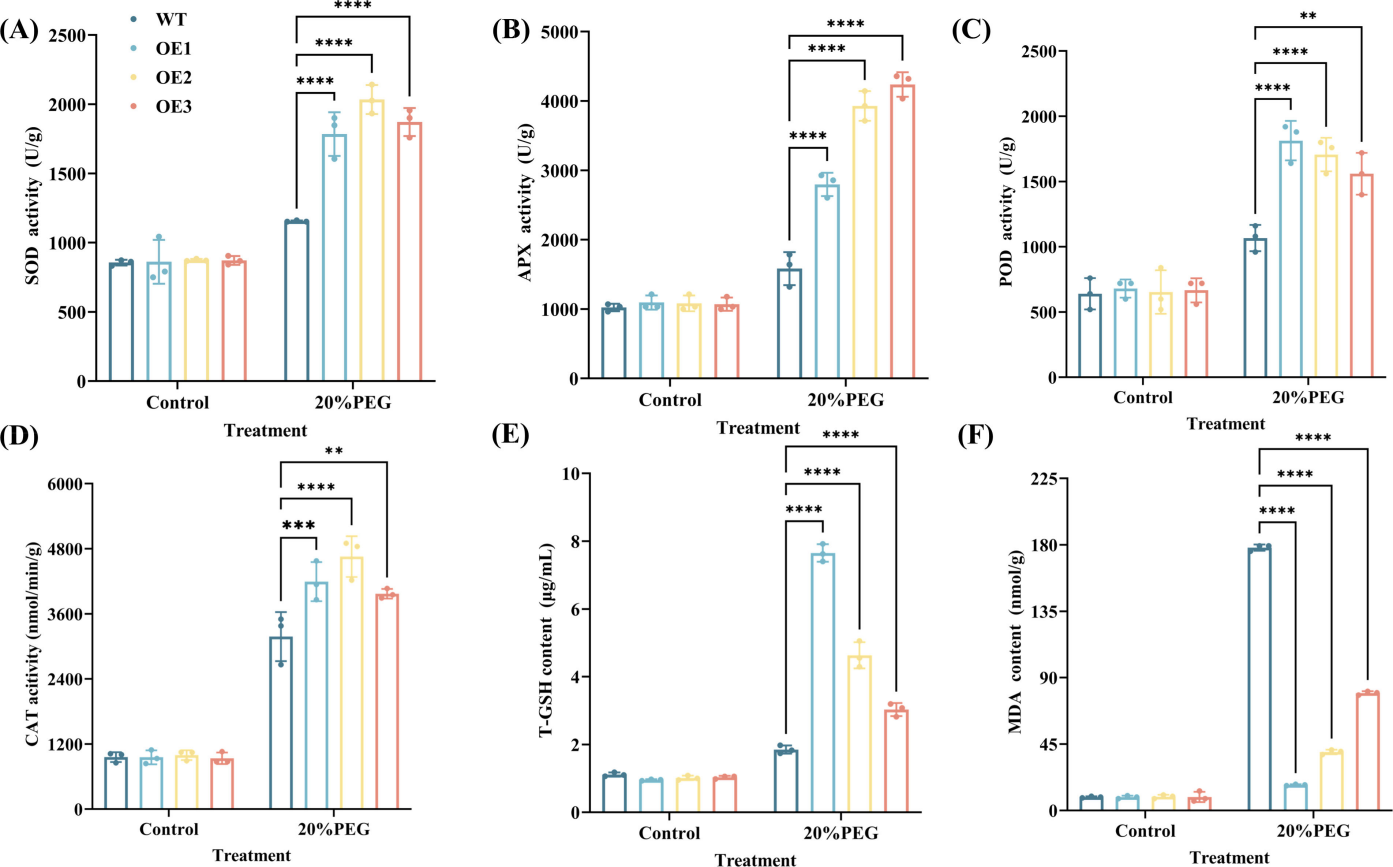
Effects of 20% PEG-induced drought stress on the antioxidant capacity of *CYP71AJ49*-overexpressing transgenic *Arabidopsis*. **(A)** SOD activity; **(B)** APX activity; **(C)** POD activity; **(D)** CAT activity; **(E)** T-GSH content; **(F)** MDA content. The vertical error bars in the figures denote means ± SD (n = 3) and asterisks indicate statistically significant (**p < 0.01; ***p < 0.001; ****p < 0.0001 based on Tukey’s HSD test).

In standard conditions, there were no marked differences observed in antioxidant enzyme activities (SOD, APX, POD, and CAT) or contents of T-GSH and MDA between WT and CYP71AJ49-OE plants. After 10 days of treatment with 20% PEG, both groups showed significantly elevated in antioxidant enzyme activities and T-GSH and MDA levels compared to the control (**Figure 7**). Moreover, under drought stress, CYP71AJ49-OE plants exhibited significantly higher antioxidant enzyme activities and T-GSH content, along with significantly lower MDA content, compared to WT. Specifically, line OE-1 showed the highest increase in POD activity and T-GSH content, which were 1.70-fold and 4.14-fold higher than WT, respectively (**Figures 7C, E**). Line OE-2 displayed the highest SOD and CAT activities, which were 1.76-fold and 1.46-fold greater than WT (**Figures 7A, D**). Line OE-3 had the highest APX activity, measuring 2.48 times that of WT (**Figure 7B**). Meanwhile, line OE-1 showed the greatest reduction in MDA content, which was 930.35% lower than that of WT (**Figure 7F**).

### 3.8 Effect of *CYP71AJ49* overexpression on Pro and chlorophyll content under drought stress

**Figure 8:**
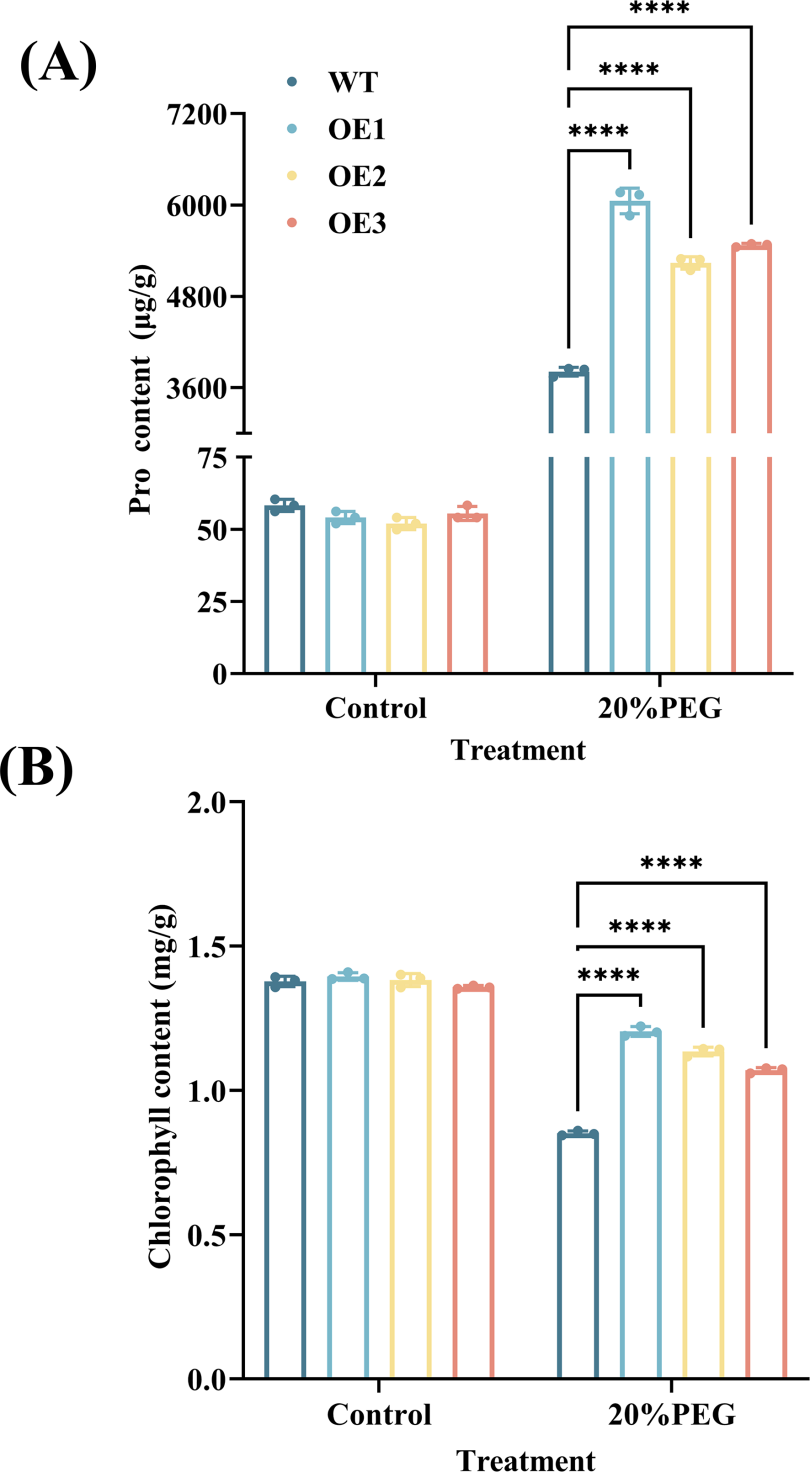
Effects of 20% PEG-induced drought stress on Pro and chlorophyll contents in *CYP71AJ49*-overexpressing transgenic *Arabidopsis*. **(A)** Pro content; **(B)** Chlorophyll content. The vertical error bars in the figures denote means ± SD (n = 3) and asterisks indicate statistically significant (****p < 0.0001 based on Tukey’s HSD test).

Under non-stressed conditions, no statistically significant differences were observed in Pro and chlorophyll content between WT and CYP71AJ49-OE plants. Following 10 days of treatment with 20% PEG, both the WT and CYP71AJ49-OE groups exhibited a significant rise in Pro content compared with their respective untreated controls, while the WT showed a significant reduction in chlorophyll content (**Figure 8**). Under drought stress, CYP71AJ49-OE plants had significantly higher Pro and chlorophyll contents than WT. In particular, line OE-1 showed increases of 58.83% in Pro and 41.52% in chlorophyll compared to WT.

### 3.9 Effect of *CYP71AJ49* overexpression on coumarin accumulation in *Arabidopsis*

**Figure 9:**
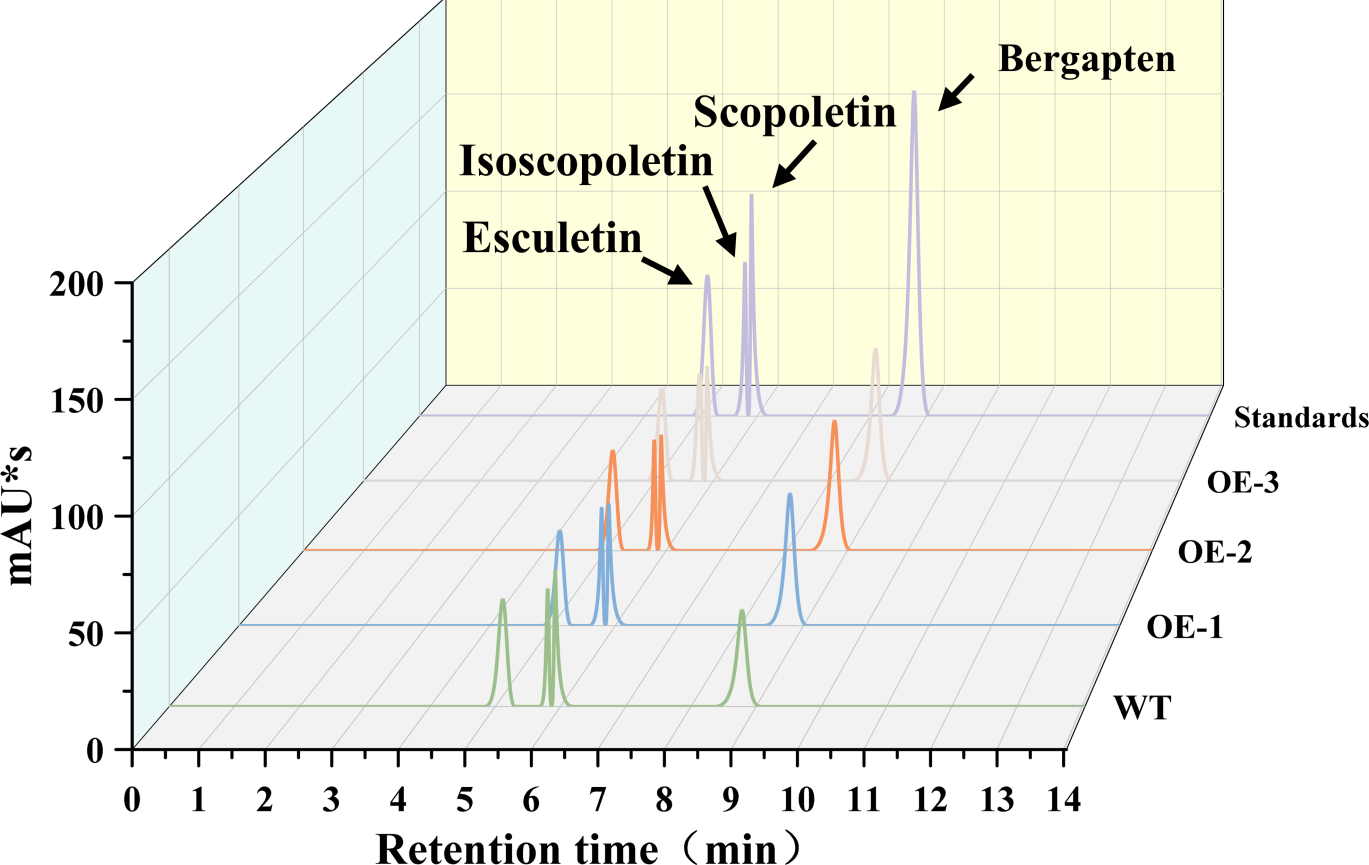
UPLC analysis of coumarin compounds in WT and *CYP71AJ49*-overexpressing transgenic *Arabidopsis*.

**Figure 10:**
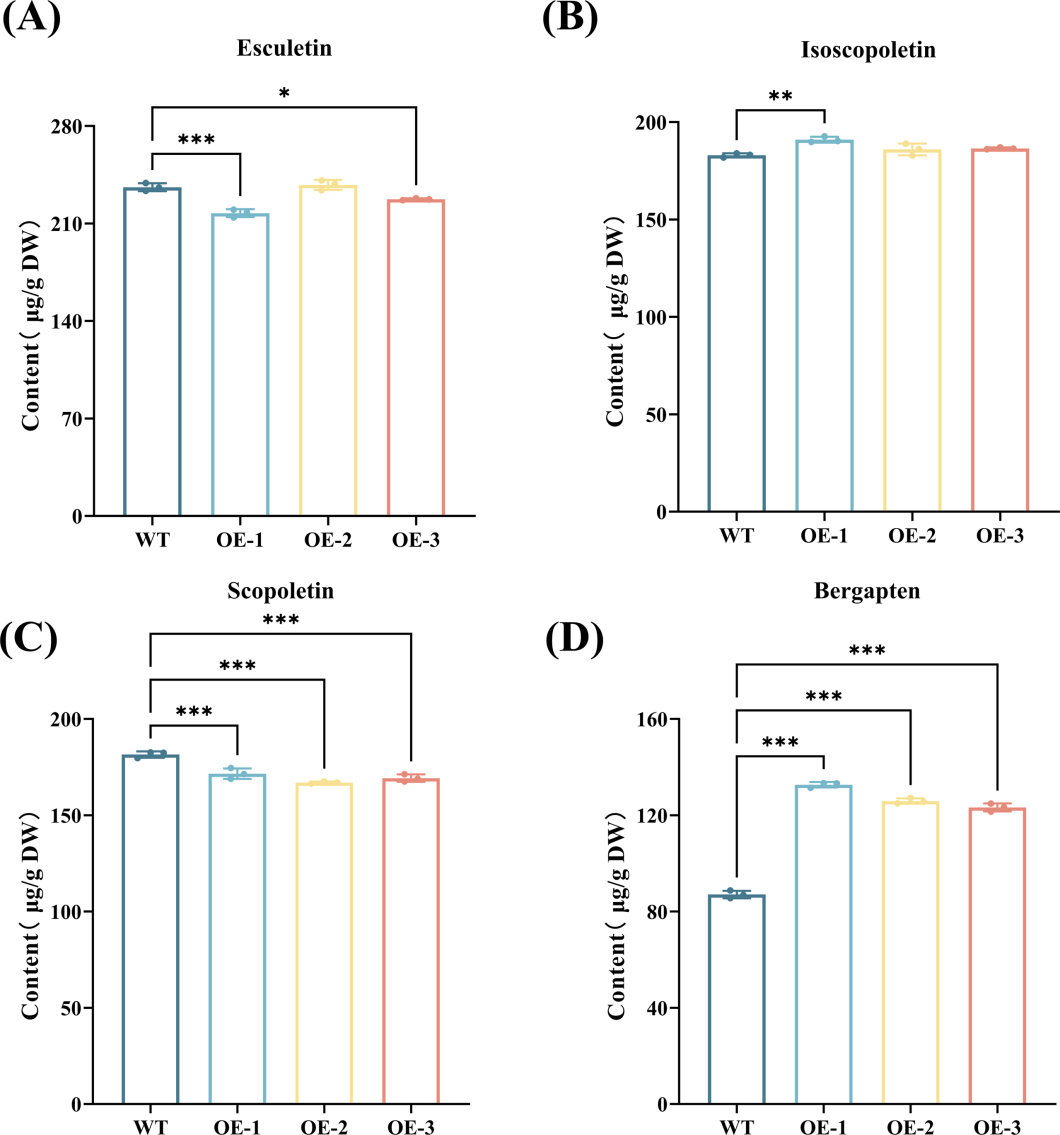
Contents of four major coumarin compounds in WT and *CYP71AJ49*-overexpressing *Arabidopsis*. **(A)** Esculetin content; **(B)** isoscopoletin content; **(C)** scopoletin content; **(D)** bergapten content. The vertical error bars in the figures denote means ± SD (n = 3) and asterisks indicate statistically significant (*p < 0.05; **p < 0.01; ***p < 0.001 based on Tukey’s HSD test).

To evaluate the impact of *CYP71AJ49* overexpression on coumarin accumulation, we measured the content of coumarins in WT and CYP71AJ49-OE plants (**Figure 9**). The results showed that scopoletin content decreased, whereas bergapten content increased significantly in CYP71AJ49-OE plants compared to WT (**Figure 10**). Line OE-1 exhibited the most pronounced changes: scopoletin decreased by 5.81%, while bergapten increased by 52.42% (**Figures 10C, D**) relative to WT.

## 4 Discussion

With the increasing severity of drought stress due to global warming, drought has become a major factor limiting plant growth and development. Improving drought resistance through genetic engineering has thus become a key research emphasis (Sato et al., 2024). The CYP71 subfamily, part of the CYP450 superfamily, is one of the largest CYP450 subfamilies in plants and serves a critical function in the synthesis and accumulation of plant secondary metabolites (Hansen et al., 2021). Previous research has demonstrated that *CYP71AJ49*, a key gene encoding a critical enzyme in the coumarin biosynthesis pathway of *P. praeruptorum*, exhibits significant alterations in transcriptional levels under abiotic stresses, including ultraviolet radiation, low temperature, and high temperature. These findings suggest a potential role for *CYP71AJ49* in regulating plant responses to adverse environmental conditions (Jian et al., 2020). In the present study, we found that overexpression of *CYP71AJ49* significantly enhanced drought tolerance in *Arabidopsis* under PEG-induced drought conditions. To elucidate the role of the *CYP71AJ49* gene in the drought stress response, a comprehensive analysis was conducted on the differences in transcriptomic profiles, stress-responsive gene expression, antioxidant enzyme activities, and the accumulation of osmoprotectants between WT and overexpression lines following a 10-day treatment with 20% PEG.

Phenotypic changes are the direct reflection of plants’ ability to adapt to adverse conditions, while physiological and biochemical indicators uncover the patterns of life activities and mechanisms of environmental adaptation during this adaptation process. Our study showed no significant phenotypic variations between CYP71AJ49-OE and WT plants under normal growth conditions, indicating that *CYP71AJ49* does not affect plant development in the absence of stress. Under drought treatment, however, CYP71AJ49-OE lines showed less severe damage symptoms compared to WT, visually confirming the positive role of *CYP71AJ49* in enhancing drought tolerance (Sahoo et al., 2022). Subsequent physiological measurements revealed that under drought stress, CYP71AJ49-OE plants had significantly greater root length, fresh weight, water content, and chlorophyll content under drought stress, along with a significantly reduced water loss rate in detached leaves. There is mutual verification among these indicators. To illustrate, a more developed root system enhances water acquisition from deeper soil, which further supports higher leaf water retention and decreases water loss associated with transpiration. These outcomes also match the drought resistance improvement seen in other relevant research (Gao et al., 2020; Raza et al., 2022; Hwang et al., 2023).

We conducted an in-depth analysis of DEGs between WT and CYP71AJ49-OE plants. GO and KEGG enrichment analyses that DEGs were significantly enriched in carbohydrate metabolism, as well as hydrolase activity, hydrolyzing O-glycosyl compounds, and a large number of these DEGs were linked to starch and sucrose metabolism pathways. This finding aligns with previous reports (Ji et al., 2023; Li et al., 2023; Fu et al., 2024) and suggests that *CYP71AJ49* may enhance drought tolerance by activating carbohydrate metabolism and hydrolytic enzyme expression. Moreover, the temporal expression patterns and expression levels of stress-responsive genes critically determine a plant’s capacity to adapt to adverse conditions, particularly those genes involved in ion homeostasis, antioxidant defense, and osmotic adjustment (Abdel-Ghany et al., 2020; Cao et al., 2024). In this study, genes related to ion homeostasis (*AtKT1*, *AtNHX1*, *AtAVP1*), antioxidant enzymes (*AtMnSOD*, *AtPOD*, *AtAPX1*), and Pro synthesis (*AtP5CS2*) showed consistent expression trends, with higher transcript levels in CYP71AJ49-OE lines than in WT across time points. These transcriptional changes closely correspond to the physiological changes we previously measured. Upregulation of *AtKT1*, *AtNHX1*, and *AtAVP1* suggests that *CYP71AJ49* helps improve ion homeostasis under drought stress (Quintero and Blatt, 1997; Wang et al., 2007). Similarly, increased expression of *AtMnSOD*, *AtPOD*, and *AtAPX1* indicates enhanced ROS-scavenging capacity, thereby alleviating oxidative damage (Yang et al., 2015; Lazzarotto et al., 2021). Furthermore, the elevated expression of *AtP5CS2* and consequent Pro accumulation in CYP71AJ49-OE plants imply that *CYP71AJ49* may contribute to osmotic adjustment under drought conditions.

Drought stress leads to water deficit, disrupts cellular ion homeostasis, induces excessive ROS production, and causes oxidative damage or even cell death (Samanta et al., 2024). To mitigate these effects, plants often enhance their antioxidant defense systems to reduce ROS accumulation (Cai et al., 2015; Shivhare and Lata, 2019). In this study, CYP71AJ49-OE plants exhibited higher activities of antioxidant enzymes (SOD, APX, POD, and CAT) and elevated levels of the antioxidant metabolite T-GSH, enabling more efficient ROS scavenging, protecting cellular membranes, proteins, and nucleic acids from oxidative damage, and alleviating impairment to the photosynthetic system. This helps explain why the CYP71AJ49-OE lines were able to maintain higher chlorophyll content and fresh weight when exposed to drought. This trend is also consistent with the findings in *CsCYT75B1*-overexpressing *Arabidopsis* under drought stress (Rao et al., 2020). Concurrently, a notable decrease in MDA content, which serves as a well-established biomarker of membrane lipid peroxidation, reflects improved cellular membrane integrity in the CYP71AJ49-OE plants. This result is consistent with their reduced phenotypic damage observed in transgenic lines under drought conditions, including higher chlorophyll content, water content, and lower leaf water loss rate. These results demonstrate that *CYP71AJ49* enhances drought tolerance by boosting antioxidant capacity and alleviating oxidative stress (Zhang et al., 2019a; Li et al., 2025), which is further corroborated by the upregulation of antioxidant enzyme genes in transgenic lines.

Additionally, secondary metabolites such as coumarins play important roles in plant stress responses. Derived from the phenylpropanoid pathway, coumarins are known for their antioxidant, antimicrobial, and anti-inflammatory properties (Younes and Mustafa, 2024; Zaynab et al., 2024). In this study, overexpression of *CYP71AJ49* specifically increased the content of bergapten, consistent with the role of the CYP71 family in modifying phenylpropanoid compounds (Bernhardt, 2006). This suggests that the psoralen synthase encoded by *CYP71AJ49* may catalyze a key step in the bergapten biosynthesis pathway. For instance, Munakata et al. (2016) reported that bergapten biosynthesis often involves O-methylation of psoralen. It is noteworthy that scopoletin content slightly decreased, whereas isoscopoletin increased mildly in transgenic plants compared to WT. Given that these compounds are isomers, this shift may indicate a regulatory role of *CYP71AJ49* in coumarin hydroxylase activity (Xu et al., 2016). Furthermore, the alterations in coumarin content and the enhanced drought resistance exhibited by transgenic *Arabidopsis* also intimate that bergapten and isoscopoletin fulfill significant functions in plant responses to drought stress.

## 5 Conclusion

This study demonstrates that *CYP71AJ49* significantly enhances drought tolerance in *Arabidopsis* through multiple mechanisms. The gene enhances drought resistance in plants by activating carbohydrate metabolism and related hydrolase pathways, upregulating the expression of stress-responsive genes, improving antioxidant enzyme activities, and promoting the accumulation of osmoprotectants. These mechanisms collectively alleviate the excessive accumulation of ROS induced by drought stress. Additionally, overexpression of *CYP71AJ49* altered the accumulation of coumarin compounds in *Arabidopsis*. These findings not only expand our understanding of the functional roles of CYP450 genes in plant stress responses but also provide a valuable candidate gene and theoretical foundation for engineering high-resilience crops through genetic approaches.

## Data Availability

The datasets presented in this study can be found in online repositories. The names of the repository/repositories and accession number(s) can be found in the article/**Supplementary Material**.
