# Supplementary material for: Functional genomic investigation of CYP71AJ49 in Peucedanum praeruptorum Dunn: Agrobacterium-mediated overexpression reveals dual roles in drought adaptation and coumarin biosynthesis
Source: Front Plant Sci. 2025 Dec 17;16:1707087. doi: 10.3389/fpls.2025.1707087 (PMC12753957; doi:10.3389/fpls.2025.1707087)
Supplement: Supplementary file 1 [file DataSheet1.docx]

Supplementary Material

# Supplementary Figures

**Supplementary Figure S1** Sequence comparison of *CYP71AJ49* gene fragment

**
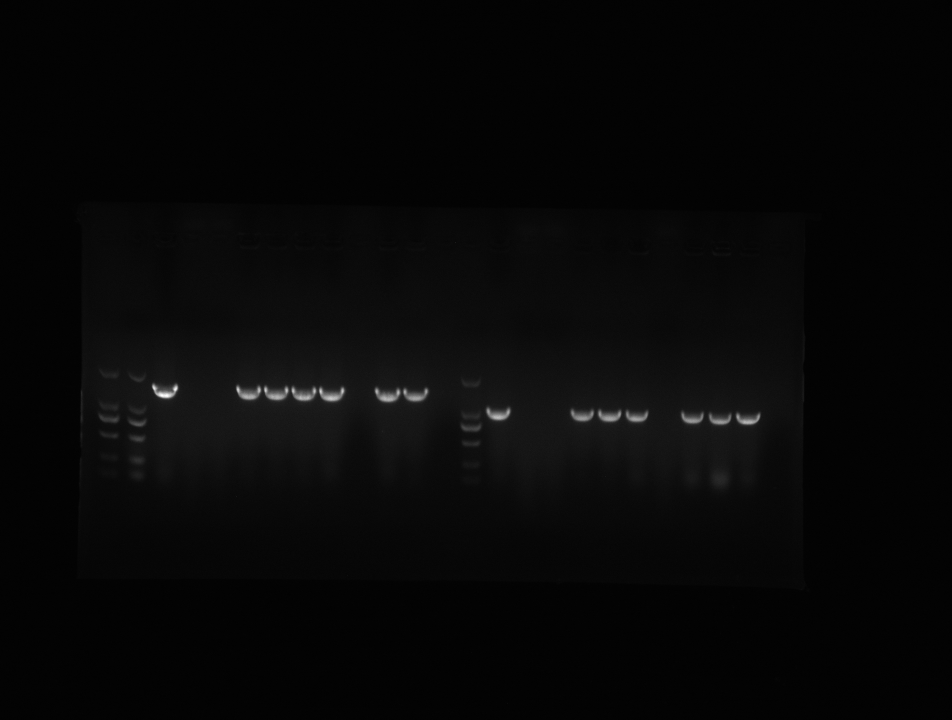
**

**Supplementary Figure S2.** Original image of FIGURE 1 gel without shearing

# Supplementary Tables

**Supplementary Table S1.** Primer sequences used for PCR amplification

| **Primer names** | **Forward primer (5'–3')** | **Reverse primer (5'–3')** | **usage** |
| --- | --- | --- | --- |
| *CYP71AJ49* | ATGAAGATGCTGGAGCAATATC | TCAAACATGTGGTGTGGCA | RCR |
| InF-*CYP71AJ49* | GGAGAGGACAGGGTACCCGGGGATCCATGAAGATGCTGGAGCAATA | ACCATGGTACTAGTGTCGACTCTAGAAACATGTGGTGTGGCAAC | In-Fusion Cloning |
| *AtACT2* | GTCGTACAACCGGTATTGTG | GAGCTGGTCTTTGAGGTTT | qRT-PCR |
| *AtKT1* | GAGGAGAGTGCATATCGAGACAAGTTC | GCAGGACGGATGTTGGGTTCAC | qRT-PCR |
| *AtNHX1* | ATGTTGGAATGGATGCCTTGGAC | CGGAAAGACGAACGCTGCTC | qRT-PCR |
| *AtAVP1* | GCAGGCAGTTCAACACCATCC | GCATGACAAGGCAACCAGGAG | qRT-PCR |
| *AtMnSOD* | CACCACCAGACTTATGTCACTCAG | CCTCCGCCGTTGAACTTGATG | qRT-PCR |
| *AtPOD* | GCATTTGTGGAGGCAATGAATAGGATG | CATGGAGCAGAGAGTTGGAGTTCAC | qRT-PCR |
| *AtAPX1* | CCGTGAGCGAAGATTACAAGAAGG | ACAATCGAAAGTTCCAGCAGAGTG | qRT-PCR |
| *AtP5CS2* | TCACCACGAGTACAGTTCCAAGG | CTACTTCACTATCTTCCGTCACTATGC | qRT-PCR |

**Supplementary Table S2.** The GenBank or RefSeq of stress-responsive *Arabidopsis* genes

| **Gene** | **GenBank/RefSeq** |
| --- | --- |
| *AtACT2* | NM_112764.4 |
| *AtKT1* | NM_128222.6 |
| *NHX1* | NM_122597.3 |
| *AVP1* | NM_101437.5 |
| *AtMnSOD* | NM_115493.4 |
| *AtPOD* | AF339700.1 |
| *AtAPX1* | NM_001123772.2 |
| *AtP5CS* | NM_115419.5 |

**Supplementary Table S3.** Gradient elution program

| **Time (min)** | **Mobile phase B (%)** |
| --- | --- |
| 0-2 | 5 |
| 2-5 | 5-40 |
| 5-7 | 40-45 |
| 7.5-8 | 45.5-46 |
| 8-9 | 46-46.5 |
| 9-10 | 46.5-50.2 |
| 10-10.5 | 50.2-50.3 |
| 10.5-11 | 50.3-50.4 |
| 11-17 | 50.4-55 |
| 17-22 | 55-80 |
| 22-25 | 80-100 |
| 25-27 | 100 |
| 27-29 | 100-5 |
| 29-30 | 5 |

**Supplementary Table S4.** Summary of read alignment statistics to the reference genome

| **Sample** | **Clean Reads** | **Total Mapped** | **Multiple Mapped** | **Uniquely Mapped** | **Map to sense strand** | **Map to antisense strand** |
| --- | --- | --- | --- | --- | --- | --- |
| OE1_1 | 41054886 | 40642321 (99.00%) | 869803 (2.14%) | 39772518 (97.86%) | 20321489 (50.00%) | 20320832 (50.00%) |
| OE1_2 | 48034044 | 47432809 (98.75%) | 1020850 (2.15%) | 46411959 (97.85%) | 23716991 (50.00%) | 23715818 (50.00%) |
| OE1_3 | 44888538 | 44160166 (98.38%) | 940672 (2.13%) | 43219494 (97.87%) | 22080573 (50.00%) | 22079593 (50.00%) |
| OE2_1 | 48500448 | 47787052 (98.53%) | 1022832 (2.14%) | 46764220 (97.86%) | 23893949 (50.00%) | 23893103 (50.00%) |
| OE2_2 | 45890934 | 45163206 (98.41%) | 904114 (2.00%) | 44259092 (98.00%) | 22582014 (50.00%) | 22581192 (50.00%) |
| OE2_3 | 50418178 | 49574432 (98.33%) | 1031260 (2.08%) | 48543172 (97.92%) | 24787318 (50.00%) | 24787114 (50.00%) |
| OE3_1 | 55535050 | 54589440 (98.30%) | 1174659 (2.15%) | 53414781 (97.85%) | 27295025 (50.00%) | 27294415 (50.00%) |
| OE3_2 | 58729608 | 57942119 (98.66%) | 1255462 (2.17%) | 56686657 (97.83%) | 28971716 (50.00%) | 28970403 (50.00%) |
| OE3_3 | 58402760 | 57327645 (98.16%) | 1257997 (2.19%) | 56069648 (97.81%) | 28664088 (50.00%) | 28663557 (50.00%) |
| WT1 | 42065564 | 41783986 (99.33%) | 860631 (2.06%) | 40923355 (97.94%) | 20892422 (50.00%) | 20891564 (50.00%) |
| WT2 | 57884830 | 57543717 (99.41%) | 1202878 (2.09%) | 56340839 (97.91%) | 28772009 (50.00%) | 28771708 (50.00%) |
| WT3 | 52728822 | 52371382 (99.32%) | 1113688 (2.13%) | 51257694 (97.87%) | 26186218 (50.00%) | 26185164 (50.00%) |
